# Integrating Scientific English into Biological Sciences PhD Programs in Developing Countries: Strategies from Trainees and Mentor

**DOI:** 10.1155/2019/3807951

**Published:** 2019-03-03

**Authors:** Camila H. Coelho, Gaspar E. Canepa, Gunjan Arora, Patrick E. Duffy

**Affiliations:** 1 Laboratory of Malaria Immunology and Vaccinology, National Institute of Allergy and Infectious Diseases, National Institutes of Health, 5640 Fishers Lane, Rockville, MD 20852, USA; 2 Laboratory of Malaria and Vector Research, National Institute of Allergy and Infectious Diseases, National Institutes of Health, 12735 Twinbrook Parkway, Rockville, MD 20852, USA; 3 Laboratory of Immunogenetics, National Institute of Allergy and Infectious Diseases, National Institutes of Health, 5625 Fishers Lane Rockville, MD 20852, USA

## Abstract

Successful researchers in the biological sciences communicate their work to a global audience and must do so in English to be widely recognized and cited. This applies equally to scientific talks, posters, and published articles; thus, scientific English must be prioritized in nonnative English-speaking (NNES) academic institutions to prepare their trainees for successful careers. Here, we propose strategies for integrating scientific English into PhD programs operating in NNES countries. Many graduate students from NNES countries strive for an international career and encounter English as an important barrier. Based on our own experiences as NNES postdoctoral fellows at a US institution, or as a US mentor of these trainees, we contend that conventional learning processes at home institutions do not sufficiently prioritize scientific English as the medium for regular discussions of laboratory-generated data. Principal investigators, mentors, and supervisors are key in promoting English language usage as a structured component of PhD training. If these stakeholders routinely integrate English training and education within the research laboratory program, graduates will be equipped to pursue international academic careers. The ideas presented here are intended for NNES PhD students (and their mentors) who seek an international scientific career in the biological sciences.

## 1. Introduction

Research into biological sciences is a global endeavor. According to the PubMed database, more than 5,000 journals publish content on biological and medical sciences, and more than 1.2 million articles were published in 2017. In one study, the “citation share” of Argentina, Brazil, Chile, China, India, Mexico, Poland, and Russian Federation for the 2005–2009 period was found to be 0.31%, 0.95%, 0.21%, 3.59%, 1.21%, 0.36%, 0.73%, and 0.83%, respectively [1]. In comparison, during the same period, the citation score from the US and EU-15 countries was 29.35% and 21.05%, respectively. Interestingly, international collaborations contribute to a large percent of papers from Argentina (45%), Brazil (26%), Chile (56%), China (22%), India (18%), Mexico (43%), Poland (33%), and the Russian Federation (32%) [1].

Articles not published in English are less read and less cited, and impact factors are higher for articles published in English [2]. Articles on Parasitology, Immunology, Microbiology, or Neuroscience, for example, are published and discussed by thousands of researchers worldwide [3], and PhD students must read publications in high-impact journals to update their knowledge of the scientific field [4, 5]. In institutions based in countries where English is the second language (nonnative English speaking (NNES) countries), students frequently use translation tools to read articles in their native language, to present a seminar, or to write a dissertation, rather than reading manuscripts in their original English. Although there are exceptions such as former British colonies in India or Africa, for example, in low- and middle-income countries (LMICs), reading and comprehending an article in English is often not an option due to inadequate resources or education [6, 7]. English classes in public schools in LMICs can be poorly taught [8, 9], and while some level of English is required for admission to the majority of graduate programs in NNES countries, students are sometimes admitted with only a modest ability to comprehend and converse in English [10].

Meanwhile, not all professors or PhD supervisors have sufficient English mastery to discuss science in a nonnative tongue [11], compounding the problem for trainees. Academic institutions sometimes seek to promote their original/native language in universities [12–14], and some countries have developed policies to increase access to higher education for disadvantaged populations [15–17]. Consequently, more people pursuing higher education lack a background in English [18–20]. Authors from NNES countries may receive more criticism about language than scientific content for manuscript they submit for review, to the detriment of the quality of the final paper [21]. These realities emphasize the need for new approaches to encourage English mastery in PhD programs worldwide.

We believe that new and effective institutional policies promoting English communication skills are needed to change the landscape for future generations of trainees. An immediate action plan will help students enrolled in PhD programs where English is not yet prioritized. As former PhD students from developing countries who are now being trained in the USA, and as a mentor of young investigators from NNES countries, we contend that students from such countries are at a significant linguistic disadvantage compared to those from English-fluent countries. Learning English in a research environment entails unique challenges: in addition to studying and performing research on complex subjects, trainees must learn the content in a nonnative language.

**FIGURE 1:**
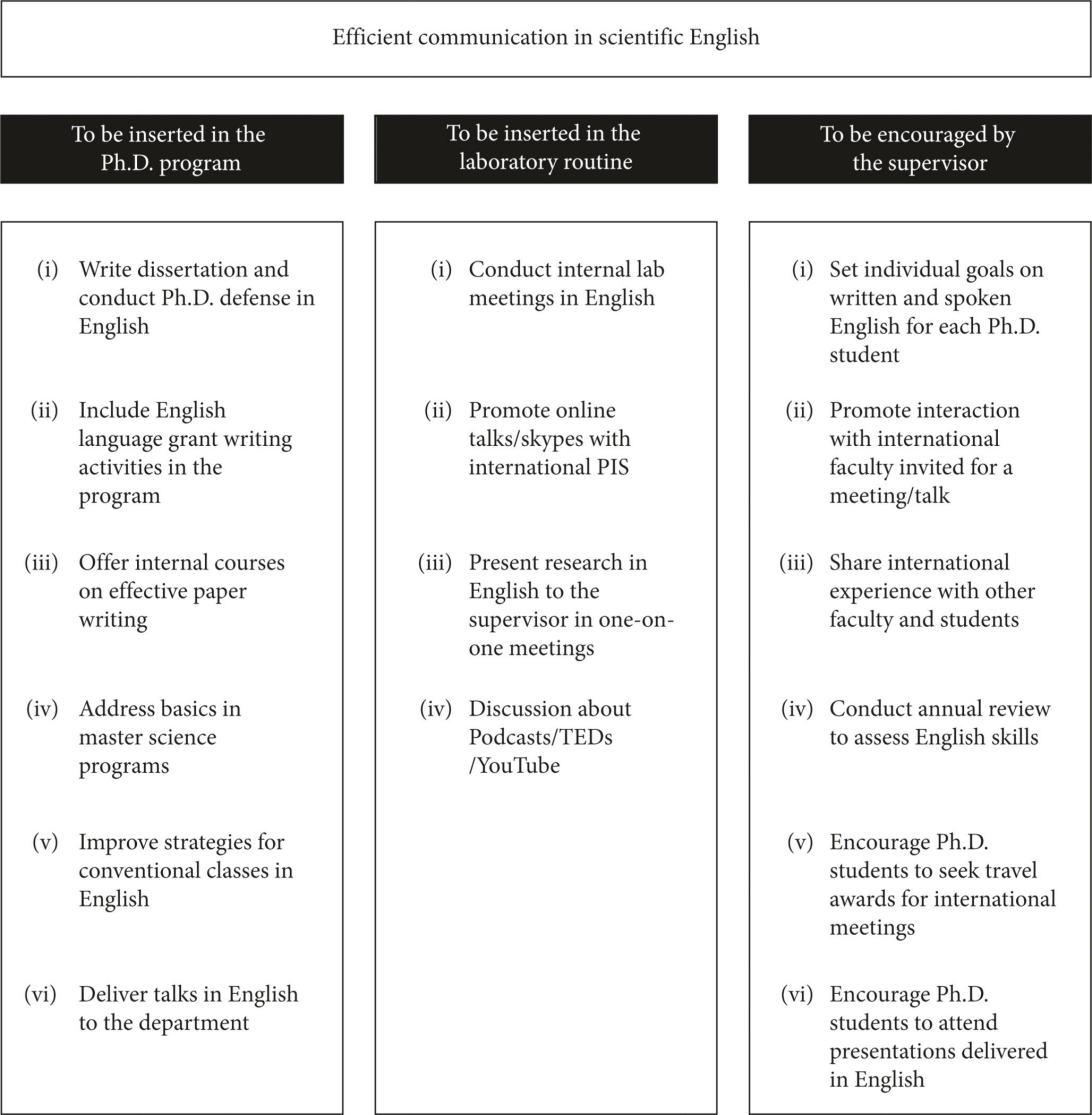


Here, we discuss strategies to integrate English language studies into routine laboratory activities for those who want to master scientific English (Figure 1). We propose approaches for students and supervisors that prioritize training in English communication in PhD programs in NNES countries.

## 2. Strategies to Promote Scientific English in PhD Programs in NNES Countries

### 2.1. Writing Dissertation and Conducting PhD Defense in English.

Writing a dissertation in English, when it is not the student’s first language, is a difficult task [22, 23]. Even in their original language, students must prepare themselves for the writing phase in a clear and comprehensive manner. Supervisors and students should consider the possibility of writing the dissertation in English, and this discussion can start upon acceptance to a PhD program. On average, a PhD program lasts from four to five years, a sufficient amount of time to execute a plan for an English language dissertation, especially if the supervisor implements other strategies to integrate English within the department and laboratory. Further, completion of a dissertation in a nonnative language represents a significant achievement and can be appropriately recognized with academic credit.

Naturally, some faculty and institutions may understandably object to the idea that no dissertations will be written in the native language. Scientific knowledge must be accessible to all populations, and we cannot simply ignore the existence of native culture and language. In addition, reading in a native language can offer a different paradigm to the reader [24]. The option of writing the dissertation in the original language must be decided in the local post-graduation program; if the university or the program does not agree to waive the requirement for a native language dissertation, perhaps English could be added as second version [25].

A dissertation written in English provides many advantages for the student, the supervisor, the program, and the country. (1) The student will hone his/her ability to write in English and prepare scientific articles for submission to premiere international journals; (2) when applying for a postdoctoral fellowship or a job opportunity abroad, the student can share the dissertation with other researchers; (3) when uploaded to the Internet, the dissertation can be accessed and read by many people overseas, creating opportunities for collaboration or discussions regarding the study; and (4) the university, postgraduate program, and supervisor equally gain visibility and stature when dissertations completed under their supervision are disseminated globally.

### 2.2. Including English Language Grant Writing Activities in the Program.

Most students lack an understanding of the grant writing process before finishing their PhD. To prepare students for writing successful grants, investigators should allow and encourage students to apply for small grants. A record of successful grant awards, no matter how small, can prepare trainees for future success as independent investigators. This exercise will also help supervisors to assess and improve a student’s ability to communicate research ideas effectively. Investigators can provide feedback to improve language and scientific writing skills at an early career stage. Students that excel in communication skills will be advantageous for their group/lab, and therefore benefit the supervisor and department.

### 2.3. Offering Internal Courses on Effective Paper Writing.

Writing effective abstracts and research papers is a learned skill. Inexperience hinders even diligent students from publishing their papers in high-impact scientific journals. Institutions should offer mandatory training courses that teach students how to write a research article. Emphasis should be placed on how to communicate effectively in scientific English. Institutions are also primarily responsible for training students to perform literature reviews and avoid plagiarism in science. Other key topics are scientific vocabulary, grammatical rules, style guides (MLA, APA, and Chicago), and citation of relevant and high-quality references. We believe that the department head or PhD program director could foster this initiative, contacting international scientific societies or publishing organizations to acquire funding or personnel for these activities.

### 2.4. Introducing English Emphasis in Master of Science Programs.

Master of Science degree programs should incorporate courses on effective scientific communication, ideally led by scientists from national or international institutions with experience in international communication and publication [26]. A focused course at the university level will ensure that students recognize the importance of English skills before launching their research careers.

### 2.5. Improving Strategies for Conventional Classes in English.

This strategy is already employed by many PhD programs, but the effectiveness varies widely [27]. In our experience, these are typically conventional classes where the professor (usually a foreign professor invited for the class) explains the content with little, if any, interaction or participation by the students. While this strategy promotes listening in English, the overall objective is best achieved by encouraging the student to ask questions and interact with the professor. Importantly, some universities limit enrollment in this type of class to students with existing fluency in English. This practice is exclusionary and should not be encouraged. Students with less knowledge in English must be gradually inserted into the context of English discussions. In addition, we believe that the creation of an English interest group in a PhD program could motivate students that share a common goal of improving scientific English discussions. Such an English club or interest group would be effective if fostered by an institutional mentor to follow overall progress of the activities.

### 2.6. Delivering Talks in English to the Department.

In addition to regular English communication within the laboratory, communications and presentations to other colleagues and faculty in the department are fundamental to prepare a student for international meetings and career opportunities. Some PhD programs in NNES countries schedule weekly or monthly meetings where PhD students present their research to the entire department or institute in their native language. These programs offer a good opportunity to insert a new strategy for presentations in English. The aforementioned guidelines for English communication in the laboratory should be observed here as well, so the student is encouraged and not intimidated from communicating in English.

### 2.7. Conducting Internal Lab Meetings in English.

This approach is a mid- to long-term goal after the student is accepted into the PhD program. In developing countries, most students are not equipped to deliver a talk in English. To overcome this, the supervisor should establish a deadline for the student’s first talk in English. Additionally, the format for the talk should put the student at ease, for example, prohibit destructive criticism during the presentation and reserve any feedback on English language usage until after the presentation.

Presenting research in English becomes easier once English is established as a norm in the laboratory. To achieve total immersion and accelerate progress, the research leader can set a policy for all internal talks to be delivered in English for continuous skill development.

### 2.8. Promoting Online Talks and Video Conferences with International Principal Investigators.

Students should learn to communicate effectively with peers and collaborators. Students from NNES countries can request informational interviews with scientists working in their research area. Such interactions can reduce language barriers and overcome natural inhibitions to interact with peers. Furthermore, these interactions will help them communicate more effectively in their future endeavors and contribute to an improved standard of science in these countries. This strategy can not only work at the individual group level but can also be promoted through institutional collaborations.

### 2.9. Presenting Research in English to the Supervisor in One-on-One Meetings.

This is a feasible approach if the supervisor has some fluency in English. The mentor may ask his/her students to present data in English, encouraging them to develop proficient speaking and writing skills. Even basic steps such as generating a graph with English axes are helpful for learning the language. Additionally, some data are generated in English by default (Proteomics, Metabolomics, RNAseq, etc.) because most database software packages are developed in English.

### 2.10. Accessing Content from Podcasts/TED/YouTube.

In the era of social networking and massive open online courses (MOOCs), new tools are available for students to hone their English language skills. Science-based podcasts can familiarize students with effective English communication and allow them to train at their own pace. Further, many MOOCs are free (e.g., Coursera and edX) and help students identify large groups with similar interests. Another widely popular and freely available resource is YouTube/TED/Twitter. YouTube/TED has numerous lectures and course materials related to scientific English that can be immensely useful for students.

### 2.11. Setting Individual Goals on Written and Spoken English for Each PhD Student.

Experienced mentors are generally able to recognize students with strong research skills versus those that require additional mentorship to achieve their objectives. In a similar fashion, we propose that mentors identify students at different levels of English mastery. Subsequently, the supervisor and student can establish a customized timeline and plan for each student to practice English communication using the strategies discussed below.

### 2.12. Promoting Interactions with International Faculty Invited for a Meeting/Talk.

One strategy to develop English skills is to offer interactions with international faculty invited for a meeting or talk. Any direct interaction will be useful to emphasize the importance of communicating about science in English. An international conference that has substantial numbers of participants from NNES countries can organize sessions where early career scientists interact with conference organizers and faculty. This provides an opportunity for students to develop effective verbal communication and networking skills.

### 2.13. Sharing International Experiences with Other Faculty and Students.

This may be one of the easiest approaches to create awareness of the importance of English communication, yet researchers often fail to recognize this opportunity. Sharing international experiences with students and new faculty conveys the value as well as the feasibility of English fluency for those working in science. Students should recognize that the most influential researchers in the department have international experience and recognition. It is important for students to hear from senior colleagues or peers that have had the opportunity to train abroad, which inspires them to recognize that they can also share their research globally. This empowers students to believe that they are capable of learning English. Mentors must show their trainees that this goal is attainable and that the laboratory or PhD program will help them.

### 2.14. Conducting Annual Reviews to Assess English Skills.

After providing mandatory training at the beginning of their PhD coursework, the institution should monitor students to ensure they maintain and improve their language skills. Depending on their background and initial English language proficiency, some students may need additional resources and training to attain a sufficient level of fluency. Scientific institutions should develop mandatory annual follow-up/refresher courses that are designed to enhance or maintain language proficiency gained during initial coursework. We highlight here, however, that mandatory trainings only can be effectively implemented with institutional support.

### 2.15. Encouraging PhD Students to Seek Travel Awards for International Meetings.

Most PhD students only become aware of the immense opportunities for fellowships or travel awards in the USA or other developed countries once they obtain a position abroad. However, in the era of the Internet, any student or supervisor can access information on numerous funding opportunities and travel award applications. Notably, many awards target young scientists from developing countries.

### 2.16. Encouraging PhD Students to Attend Presentations Delivered in English.

When departments host international speakers, or when meetings with an international faculty convene in the country, students should be encouraged or required to attend. It is important to discourage students from using translation devices and to encourage them to pose a question to the speaker. Following talks or meetings in English, students should submit reports or lead discussions to share the knowledge they gained.

## 3. Conclusions

In this paper, we proposed strategies to promote scientific skills based on our experiences as former PhD students from NNES countries and as a mentor of these young scientists. These strategies and tools can motivate and empower students to acquire the English fluency necessary to communicate and succeed in science. Students should be immersed in activities requiring English usage through a structured program that requires routine practice. We believe that supervisors must increase students’ exposure to English at the outset of their career and must recognize their role as critical agents to implement effective policies throughout the academic environment.

## Abbreviations

NNES: Nonnative English speaking
LMICs: Low- and middle-income countries
MOOCs: Massive open online courses
